# Comparison of different correction formulas and measurement methods for the accurate determination of intraocular pressure after SMILE and FS-LASIK surgery

**DOI:** 10.1186/s12886-022-02620-7

**Published:** 2022-10-10

**Authors:** Zhiqing Yang, Na Miao, Lixiang Wang, Ke Ma

**Affiliations:** 1 Department of Ophthalmology, West China Hospital of Sichuan University, No. 37 Guoxue Alley, Chengdu, 610041 Sichuan Province China; 2 West China Medical School, Sichuan University, Chengdu, 610041 Sichuan Province China

**Keywords:** Intraocular pressure, Refractive corneal surgery, Non-contact tonometry, Pentacam, Topography map, Corvis ST

## Abstract

**Background:**

To compare the accuracy of non-contact tonometry, Pentacam, and corneal visualization Scheimpflug technology (Corvis ST) for the measurement of intraocular pressure (IOP) after small incision lenticule extraction (SMILE) or femtosecond laser-assisted in situ keratomileusis (FS-LASIK) surgery.

**Methods:**

A total of 49 patients (98 eyes) undergoing FS-LASIK or SMILE surgery at West China Hospital, Sichuan University from January to March 2021 were enrolled in this prospective, comparative, self-controlled study. IOP values were measured with non-contact tonometer, Pentacam, and Corvis ST before surgery and 1 month after surgery. Pentacam-derived postoperative IOP values were corrected using five correction formulas (Ehlers, Shah, Dresden, Orssengo-Pye, and Kohlhaas), while Corvis ST-derived values were corrected using a single formula.

**Results:**

In the SMILE group, no significant differences were observed between the IOP values corrected with the Shah formula before and after surgery (*t* = 0.17, *P* = 0.869), whereas postoperative IOP values corrected with the other formulas were significantly different from the corresponding preoperative measurements (*P* < 0.05). In the FS-LASIK group, postoperative IOP values corrected with the Ehlers, Shah, or Corvis ST formulas were significantly different from the corresponding preoperative IOP measurements (*P* < 0.05), but no significant differences were observed between pre- and postoperative IOP values corrected with the Dresden (*t* =  − 0.08, *P* = 0.941), Orssengo-Pye (*t* =  − 0.52, *P* = 0.604), or Kohlhaas (*t* = 1.22, *P* = 0.231) formulas.

**Conclusions:**

Pentacam’s Shah correction formula seemed to be the most appropriate method for accurately measuring postoperative IOP in patients undergoing SMILE surgery, while the Dresden, Orssengo-Pye, and Kohlhaas correction formulas of Pentacam were identified as the most reliable methods for estimating IOP in patients after FS-LASIK surgery.

**Supplementary Information:**

The online version contains supplementary material available at 10.1186/s12886-022-02620-7.

## Background

Corneal refractive surgery is used to correct the refractive errors of patients so that external objects can be clearly imaged on the retina. Small incision lenticule extraction (SMILE) and femtosecond laser-assisted in situ keratomileusis (FS-LASIK) are the two most common types of corneal refractive surgeries that can correct myopia and astigmatism with good efficacy, safety, stability, and predictability [1].

Myopia has been identified as a risk factor for primary open-angle glaucoma [2]. In addition, glucocorticoid eye drops are used for a long time during the postoperative period to control the wound healing response, which increases the risk of secondary glaucoma after SMILE and LASIK surgery. Therefore, the accurate determination of the postoperative IOP is particularly important. However, the non-contact tonometer (NCT) and the “gold standard” Goldmann applanation tonometry are both subject to changes in corneal curvature, thickness, and biomechanical parameters, which tend to underestimate postoperative IOP [3, 4]. As a result, postoperative IOP values should be corrected to accurately diagnose glaucoma caused by long-term glucocorticoid use [5].

Currently, a number of correction methods and formulas are available which take into account the corneal thickness or biomechanical parameters change to determine the postoperative IOP of surgery patients, including Pentacam-corrected IOP and Corvis ST-corrected IOP. However, no previous studies have compared their accuracy in regard to different surgical types. As determined by previous animal studies, corneal refractive surgery doesn’t cause IOP change [6]. So in this study, we used the preoperative IOP as the reference and compared it with the postoperative IOP at 1 month corrected by Pentacam and Corvis ST using different formulas to identify the optimal methods for accurately estimating IOP in myopic patients after corneal refractive surgery.

## Methods

### Participants

This is a prospective study enrolling patients with myopia or myopic astigmatism who underwent corneal refractive surgery from January to March 2021 at the Myopia Surgery Center of West China Hospital, Sichuan University. The sample sizes were calculated by G*power software (version 3.1.9.6). By setting α = 0.05, 1–0 = 0.95, and mean difference and standard deviation of IOP reduction tested by non-contact tonometer as 3 and 5 mmHg (effect size = 0.60) [7, 8], a respective sample size of 32 eyes of each type of surgery was determined to be appropriate. A total of 49 patients (98 eyes) were randomly selected with a random number table from screened patients suitable for the surgery during the recruitment period, of whom 29 patients (58 eyes) underwent SMILE surgery and 20 patients (40 eyes) underwent FS-LASIK surgery. The study was approved by the Ethics Committee of West China Hospital of Sichuan University and was performed in accordance with the declaration of Helsinki. The inclusion criteria were as follows: (1) 18–40 years old; (2) range of spherical diopter of subjective refraction: 0.00 diopter (D) to − 9.00 D; (3) range of cylinder diopter: <  − 6.00 D; (4) the refractive power remained stable (change < 0.5 D) in the past two years; (5) quit soft contact lenses for more than a week or hard contact lenses for more than a month prior to the study; (6) range of preoperative IOP measured by non-contact tonometer: 10–21 mmHg; (7) normal corneal morphology without keratoconus or corneal pannus; (8) no other ophthalmic diseases, such as cataract, glaucoma, or fundus lesions; (9) no history of eye surgery or trauma; (10) no active inflammation; and (11) no history of connective tissue disease, systemic disease, or drug allergy. Patients with presumed ocular hypertension caused by corticosteroids during early postoperative visits were excluded, which was clinically determined by abnormal elevation of IOP in early postoperative period responsive to steroid suspension.

### Measurement and correction of IOP

All patients underwent standard preoperative refractive examinations including uncorrected visual acuity, computerized refractometer refraction, retinoscopy, refraction with small pupils, slit lamp microscopy, and A-scan ultrasound. IOP in all patients was measured before surgery and one month after surgery. The same experienced investigator performed all IOP measurements and corrections with NCT (Oculus, Germany), Pentacam (Oculus, Germany), and Corvis ST (Oculus, Germany).

#### IOP estimation with NCT

NCT is a simple and fast method widely used for measuring IOP in clinics with good accuracy and repeatability, avoiding the application of topical anesthesia and the risk of cross-infection [9]. When measuring IOP, the seat and jaw rests were first adjusted to the appropriate position, and the patient sat comfortably with the forehead resting closely against the device and the head in an upright, fixed position. The patient was then asked to relax and look at the fixation lamp. Here, the IOP in each eye was measured multiple times and only corneal topographic images with a quality mark of "OK" were considered valid. The difference between each IOP measurement must be less than 3 mmHg. The mean value of three valid IOP measurements was considered as the final NCT value.

#### IOP corrected with Pentacam

Pentacam corneal topography is used to measure the corneal thickness as well as the curvature of the anterior and posterior corneal surface [10]. Here, valid measurements were obtained following the same process as during NCT readings, and the recorded data were used to calculate the corrected IOP values based on the following formulas [11]: Ehlers IOP (IOPe) = IOPmeasured + [0.071 × (545 − corneal thickness)] [12]; Shah IOP (IOPs) = IOPmeasured + [0.050 × (550 − corneal thickness)] [13]; Dresden IOP (IOPd) = IOPmeasured + [0.040 × (550 − corneal thickness)] [14]; Kohlhaas IOP (IOPk) = IOPmeasured + [(540 − corneal thickness)/71] + [(43-K)/2.7] + 0.75 [15]; and Orssengo-Pye IOP (IOPo) = Goldmann IOP /K [16].

#### IOP corrected with Corvis ST

Corvis ST has recently emerged as a novel technique for assessing the biomechanical parameters of the cornea. Briefly, a balanced air pulse is applied to the cornea and the dynamic response is recorded with an ultra-high-speed Scheimpflug camera to measure IOP and other biomechanical parameters, such as corneal thickness, corneal curvature, and morphology parameters, providing a biomechanically-corrected IOP [17]. Here, valid measurements were obtained following the same process as during NCT readings. Biomechanically-corrected IOP by using the latest version of software on Corvis ST (version 1.6B2042) was recorded and hereafter referred as bIOP. As demonstrated by previous studies, bIOP is a more accurate method for IOP determination after corneal refractive surgeris compared to noncontact IOP measurement (referred as nctIOP), which is also provided by Corvis ST but not used for analysis in our study [18, 19]. Each eye was measured multiple times and the average of three valid IOP measurements was considered as bIOP. The difference between valid bIOP measurements of the same eye should be lower than 3 mmHg.

### Surgical methods and postoperative medication

SMILE and FS-LASIK corneal refractive surgeries were performed following standard procedures without intraoperative complications by the same experienced surgeon.

After surgery, patients were given a standard regimen of postoperative topical eye drops, including (1) tobramycin eye drops (four times daily until full consumption of the prescribed dose, which was usually 2 weeks); (2) ophthalmic gel of deproteinized calf blood extract (four times daily for two weeks); (3) tobramycin/dexamethasone eye drops (four times daily for three days), then switching to loteprednol eye drops (three times daily and then once every 10 days until the end of day 30); (4) brimonidine tartrate eye drops (two times daily until full consumption of the prescribed dose, which was usually 2 weeks); and (5) artificial tears, including 0.3% sodium hyaluronate eye drops (two times daily until full consumption, which was usually 3 months), polyethylene glycol eye drops (once daily until full consumption, which was usually 3 months), and 0.1% sodium hyaluronate eye drops (once daily until full consumption, which was usually 3 months).

### Statistical analysis

Statistical analysis was performed using SPSS version 22.0 (IBM, Chicago, IL, USA). The single-sample Kolmogorov–Smirnov test was used to test whether the data follows the normal distribution. Normally distributed data were expressed as mean ± standard deviation (SD). The paired *t*-test was used to compare IOP values before and after surgery, while analysis of variance (ANOVA) was used to compare IOP values obtained using different measurement methods and correction formulas. Differences with *P* < 0.05 were considered statistically significant.

## Results

### Patient characteristics

A total of 49 subjects (98 eyes) were enrolled in the study, including 15 men (30 eyes) and 34 women (68 eyes) with an average age of 26.12 ± 5.21 (range 18–37) years. The overall preoperative refractive measurements were as the following: mean spherical diopter (− 5.01 ± 1.57) D (range − 1.75D to − 8.00D); mean cylindrical diopter (− 0.84 ± 0.74) D (range 0.00D to − 3.50D); and mean equivalent spherical diopter (− 5.37 ± 1.64) D (range − 1.75D to − 8.50D).

Of the 49 patients, 8 men (16 eyes) and 21 women (42 eyes) with an average age of 26.38 ± 5.69 (range 18–37) years underwent SMILE surgery. The mean preoperative spherical diopter was (− 4.56 ± 1.51) D (range − 1.75D to − 7.75D); the mean cylindrical diopter was (− 0.62 ± 0.50) D (range 0.00D to − 1.75D); and mean equivalent spherical diopter was (− 4.83 ± 1.54) D (range − 1.75D to − 7.75D).

The remaining patients, including 7 men (14 eyes) and 13 women (26 eyes) with an average age of 25.75 ± 4.47 (range 19–35) years, underwent FS-LASIK surgery. The mean preoperative spherical diopter was (− 5.66 ± 1.44) D (range − 2.25D to − 8.00D); mean cylindrical diopter was (− 1.16 ± 0.91) D (range 0.00D to − 3.50D); and mean equivalent spherical diopter was (− 6.16 ± 1.46) D (range − 3.25D to − 8.50D).

### Pre- and postoperative IOP

In patients undergoing SMILE surgery, NCT measurements of IOP, as well as bIOP, IOPe, IOPd, IOPk, and IOPo, differed significantly between preoperative measurements and postoperative measurements at 1 month. However, IOP correction with the Shah formula showed no significant difference before and after surgery (Table 1).

**Table 1:** Comparison of IOP measurements with different methods and correction formulations before and after SMILE surgery

Timepoint	No eyes	NCT- measured IOP (mmHg)	Corvis ST-corrected IOP (bIOP) (mmHg)	Pentacam-corrected IOP (mmHg)				
				Ehlers	Shah	Dresden	Orssengo-Pye	Kohlhaas
				(IOPe)	(IOPs)	(IOPd)	(IOPo)	(IOPk)
Preoperative	58	15.78 ± 2.20	15.82 ± 1.41	15.35 ± 2.02	15.73 ± 1.89	15.74 ± 1.88	15.50 ± 1.75	16.28 ± 1.95
1 month		11.19 ± 2.06	14.73 ± 1.48	17.19 ± 2.20	15.68 ± 1.96	14.77 ± 1.91	14.36 ± 2.45	14.45 ± 1.92
Change		-4.59 ± 2.63	-1.09 ± 1.95	1.83 ± 2.43	-0.05 ± 2.39	-0.97 ± 2.42	-1.14 ± 2.78	-1.83 ± 2.44
*t* value		13.27	4.23	-5.75	0.17	3.04	3.12	5.7
*P* value		< 0.001	< 0.001	< 0.001	0.869	0.004	0.030	< 0.001

1 mmHg = 0.133 kPa

NCT-derived IOP as well as corrected IOP by bIOP, IOPe, and IOPs revealed a significant difference between pre- and postoperative values in patients undergoing FS-LASIK surgery. However, no significant differences were found in IOPd, IOPo, or IOPk before and after the surgery. ANOVA analysis showed that the three corrected estimates IOPd, IOPo, and IOPk did not differ significantly from one another, either before or after surgery (homogeneity of variance, *P* = 0.201; *F* = 0.733, *P* = 0.483, Table 2).

**Table 2:** Comparison of IOP measurements with different methods and correction formulations before and after FS-LASIK surgery

Timepoint	No eyes	NCT- measured IOP (mmHg)	Corvis ST-corrected IOP (bIOP) (mmHg)	Pentacam-corrected IOP (mmHg)				
				Ehlers (IOPe)	Shah	Dresden	Orssengo-Pye	Kohlhaas
					(IOPs)	(IOPd)	(IOPo)	(IOPk)
Preoperative	40	15.58 ± 2.42	16.85 ± 2.10	16.40 ± 2.10	16.50 ± 2.08	16.35 ± 2.10	16.10 ± 2.22	16.40 ± 2.42
1 month	40	12.14 ± 3.18	15.33 ± 2.43	19.30 ± 3.10	17.46 ± 2.43	16.38 ± 3.00	16.39 ± 3.89	15.90 ± 3.22
Change		-3.67 ± 2.70	-1.52 ± 2.01	2.90 ± 2.92	0.96 ± 2.79	0.03 ± 2.75	0.29 ± 3.49	-0.50 ± 2.60
*t* value		8.58	4.77	-6.28	-2.18	-0.08	-0.52	1.22
*P* value		< 0.001	< 0.001	< 0.001	0.035	0.941	0.604	0.231

Taken together, these results suggest that the Shah correction formula of Pentacam seems to be most suitable for accurately determining IOP after SMILE surgery, whereas the Dresden, Orssengo-Pye, and Kohlhaas formulas are all suitable for correcting IOP after FS-LASIK surgery. As for patients undergoing corneal refractive surgery of undetermined types (FS-LASIK or SMILE), the combined data demonstrated in Table 3 suggested that both the Shah and Orssengo-Pye correction formulas could be used. However, the Shah correction formula generated a smaller difference between pre- and postoperative IOP measurements. Thus, the Shah correction may be a better choice for estimating IOP after SMILE and FS-LASIK surgery.

**Table 3:** Comparison of IOP measurements with different methods and correction formulations before and after either type of corneal refractive surgeries

Time	No. eyes	NCT- measured IOP (mmHg)	Corvis ST-corrected (bIOP) (mmHg)	Pentacam-corrected IOP (mmHg)				
				Ehlers IOP (IOPe)	Shah	Dresden	Orssengo-Pye	Kohlhaas
					(IOPs)	(IOPd)	(IOPo)	(IOPk)
Preoperative	98	15.79 ± 2.28	16.24 ± 1.79	15.78 ± 2.11	16.04 ± 1.99	15.99 ± 1.99	15.75 ± 1.97	16.33 ± 2.14
1 month	98	11.58 ± 2.60	14.98 ± 1.94	18.05 ± 2.78	16.41 ± 2.58	15.43 ± 2.53	15.19 ± 3.25	15.04 ± 2.62
Changes		-4.21 ± 2.69	-1.26 ± 1.98	2.27 ± 2.68	0.36 ± 2.60	-0.56 ± 2.60	-0.56 ± 3.16	-1.29 ± 2.58
t value		15.52	6.31	-8.38	-1.38	2.13	1.75	4.94
*P* value		< 0.001	< 0.001	< 0.001	0.17	0.035	0.084	< 0.001

## Discussion

IOP is one of the most reliable indicators for assessing glaucoma risk and the need for intervention [20]. However, there is no uniform standard for accurately determining IOP after corneal refractive surgery due to corneal ablation [21]. In this study, we used NCT and two common techniques to estimate pre- and postoperative IOP in patients undergoing SMILE and FS-LASIK surgery and compared the measurements using different correction formulas. With the preoperative IOP as the standard reference, we found that IOPs determined no significant change after surgery at 1 month in patients receiving SMILE surgery, while IOPd, IOPo, and IOPk showed no change in patients undergoing FS-LASIK surgery.

Devices commonly used to estimate IOP, such as the NCT, Goldmann applanation tonometer (GAT), and ocular response analyzer (ORA), tend to underestimate post-operative IOP values after corneal refractive surgery. For instance, it has been found that the IOP value measured by NCT after LASIK surgery decreased by (0.029 ± 0.003) mmHg for every 1 μm ablation of the central cornea. Thus, an average ablation of 15 μm per diopter of correction resulted in an IOP change of approximately 0.5 mmHg [22]. A study of 47 patients undergoing LASIK surgery found that the NCT-measured IOP decreased by (5.65 ± 1.71) mmHg after surgery compared to preoperative values [23]. In another study, the IOP measured by NCT in 93 patients undergoing LASIK surgery decreased by (5.41 ± 1.89) mmHg one month after surgery and (5.73 ± 2.03) mmHg three months after surgery [24]. A substantial decrease in GAT-derived IOP has also been described for patients undergoing FS-LASIK surgery [25]. The same trend has been reported after SMILE surgery as well. A study measuring IOP in 60 patients undergoing SMILE surgery showed that the preoperative NCT measurement decreased by (3.91 ± 1.97) mmHg and the GAT-measured IOP value decreased by (5.51 ± 2.42) mmHg three months after surgery [21].

GAT has long been recognized as the “gold standard” in IOP estimation since its introduction in 1950s [26]. As with NCT, GAT also provides an indirect estimation of IOP, which calculates IOP by the applanation force. GAT is based on the principle of applanation formula by Imbert and Fick and assumes that IOP and the force required to achieve applanation show linear relationship [27]. GAT demonstrates superior intra- and interobserver repeatability, and is mechanically more reliable compared to other IOP-measuring equipment [28]. However, just like NCT, the applanation force is also subject to changes in total corneal thickness, curvature, rigidity, and tear film variations, which can significantly influence the accuracy of GAT and appear as a particular problem in refractive surgical eyes [29]. For IOP measurements in patients undertaking corneal refractive surgeries, the accuracy of IOP measurement with GAT has been questioned. In a comparative study, the postoperative IOP by GAT in patients receiving transepithelial photorefractive keratectomy (TPRK), LASIK, and SMILE surgery at 3 month all decreased significantly (-1.78 ± 2.29 mmHg, -3.38 ± 2.76 mmHg, and -2.83 ± 2.08 mmHg, respectively). The IOP measured by GAT even demonstrated higher reduction compared to novel methods such as the dynamic contour tonometer and Corvis-ST [30].

ORA has emerged as a new type of NCT that may be less sensitive than GAT or NCT to changes in corneal biomechanical properties after LASIK surgery [25]. During ORA examination, a controlled air pulse is puffed onto the eye, and two IOP values are recorded. The mean value of the two IOP measurements is then used to simulate Goldmann IOP, while the calculated difference between the two IOP values is used to determine corneal hysteresis, corneal resistance factor, and corneal-compensated IOP. Although ORA provides more complete biomechanical data than GAT, one study found that ORA-derived IOP after FS-LASIK surgery was 0.67 ± 2.07 mmHg lower than the preoperative value [25]. In another study, ORA indicated a corneal-compensated IOP at three months after SMILE that was 2.51 ± 2.35 mmHg lower than the preoperative value [21]. These two studies suggest that biomechanical-corrected IOP by ORA may still be inaccurate for measuring IOP after corneal refractive surgery.

Clinical studies and experiments in animal models have shown that corneal refractive surgery does not lead to intraocular hypotension [6], suggesting that the reduced IOP measurements recorded after surgery need to be corrected. Pentacam is a common method to correct IOP, which takes corneal thickness into account. However, many correction formulas have been proposed and the appropriate choice of formulas is controversial. One study found the Shah correction formula is an appropriate way to correct postoperative IOP after LASIK surgery [21]. Nevertheless, two studies in which the Ehlers correction formula was used to correct IOP in 105 patients undergoing LASIK [20] or 62 patients undergoing epithelial LASIK surgery [31] showed that the difference between pre- and postoperative IOPe was not significant, indicating that the Ehlers correction formula can be used to correct IOP after either type of LASIK. Similar results were obtained in another study comparing Pentacam-corrected IOP and IOP measured by dynamic contour tonometry before and after LASIK [32].

In this study, we found that different correction formulas may be chosen in FS-LASIK and SMILE surgery groups. The difference between pre- and postoperative IOPs was insignificant, suggesting that Pentacam’s Shah formula is the most appropriate method for accurately calculating IOP after SMILE surgery. Besides, we found that the Dresden, Orssengo-Pye, and Kohlhaas formulas all generated similar IOP measurements compared to preoperative values in patients undergoing FS-LASIK. However, we did not identify the Ehlers formula as an appropriate method for correcting IOP values after FS-LASIK. The difference in the correction formula may be associated with the different corneal ablation methods, in which FS-LASIK creates a corneal flap with a femtosecond laser while SMILE extracts an intrastromal lenticule by femtosecond laser.

The various Pentacam correction formulas gave considerably different IOP values even when the formulas used a common estimated corneal thickness. This reflects the different ways that the correction algorithms account for the effects of corneal thickness on IOP. Except for the Kohlhaas formula, which takes into account both the thickness and the curvature of the anterior surface of the cornea, the other four correction formulas of Pentacam include only the corneal thickness and show the same trend in IOP evaluation: as the thickness increases, the corrected IOP value decreases. A previous study [31] defined the standard corneal thickness as 550 μm for the Dresden formula and 545 μm for the Ehlers formula, yet when the corrected IOP value changed by 1 mmHg, the corneal thickness corrected by the Dresden formula changed by 25 μm, whereas the Ehlers formula changed by 15 μm. Thus, correction algorithms may require substantially different adjustments in corneal thickness in order to correct the same IOP fluctuation.

Corvis ST has recently emerged as a more convenient and faster method than Pentacam for examining corneal biomechanics and measuring IOP. However, only a few studies have compared IOP values before and after corneal refractive surgery, reporting that IOP obtained using Corvis ST is more reliable than that measured by conventional NCT [33]. In a study of myopic patients, Corvis-derived IOP was found to be in good agreement with the GAT value [34]. However, in another study, Corvis ST was used to detect IOP of ex vivo human globes, which is manually changed by adjusting the internal inflation rig. IOP corrected by Corvis ST was found to be correlated with corneal thickness and had a large mean difference of (7.5 ± 3.2) mmHg compared to true IOP measured by the internal sensor [35]. In a previous work, IOP measured by Corvis-ST (bIOP) showed significant reduction in patients undertaking FS-LASIK (-1.21 ± 1.72 mmHg) and SMILE (-1.46 ± 1.43 mmHg) surgery at 3 months compared to preoperational values, but the change was insignificant in the TPRK group. A smaller change in b-IOP was also reported compared to NCT and GAT. This finding is consistent with the results of our study, which shows that bIOP provides a more approximate yet still significant under-estimation of post-operational IOP after SMILE and FS-LASIK surgery [30].The corneal flap or lenticule bag created during FS-LASIK or SMILE surgery can contribute to substantial changes in corneal biomechanical properties and conduction of external forces, leading to inaccurate IOP estimation, while TPRK leaves no corneal cut or flap and tends to cause smaller changes in post-operational IOP compared to pre-operational values [36, 37].

In our study, we found that preoperative Corvis-derived IOP (bIOP) differed significantly from the corresponding postoperative values after both SMILE and FS-LASIK surgery, suggesting that Corvis ST is unsuitable for measuring IOP after corneal refractive surgery. In addition, the repeatability of Corvis ST measurements was poor in our study. Nevertheless, the average differences between pre- and postoperative Corvis-derived IOP were 1.09 ± 1.95 for SMILE and 1.52 ± 2.01 for FS-LASIK, which were significantly lower than the corresponding differences with NCT.

In this study, we compared the IOP 1 month after surgery with the preoperative measurements for the evaluation of different IOP-measuring techniques and correction formulas. As the IOP after corneal refractive surgery tends to fluctuate during the early postoperative period due to surgical procedures and frequent corticosteroid use, which becomes stable after 1 month [24], we chose 1 month as the time point. With no gold standard of IOP measurements after corneal refractive surgery available, we chose the preoperative IOP values as the reference and assumed them to be unchanged 1 month after surgery. However, during this recovery period, patients were routinely treated with tobramycin/dexamethasone eye drops for three days and loteprednol eye drops for 30 days to prevent postoperative inflammation and promote wound healing. Since these formulations can increase IOP, brimonidine tartrate eye drops were also prescribed to keep the pressure under control, which may all contribute to IOP change after surgery. So future studies should measure it at three and six months after surgery, perhaps even longer, when patients are free of topical drugs. Besides, GAT as a “gold standard” in IOP evaluation was not included in our study, which is identified as a limitation. As mentioned in the previous text, corneal ablation and changes in corneal curvature, rigidity, and tear film quality all significantly contribute to IOP changes by GAT after corneal refractive surgery. Previous studies have challenged the accuracy of GAT in IOP measurement in patients undertaking corneal refractive surgeries. In addition, to prevent cross-infection of SARS-CoV-2 in patients during IOP estimation, according to the expert consensus of preventing hospitalized spreading of SARS-CoV-2 during COVID-19 pandemic in China and global recommendations, our eye center only conducted non-contact tonometer as a routine practice due to the very large amount of patient loads [38, 39]. Another limitation of our study is that IOP was not always measured at the same time of the day, which may have affected our results since IOP can change slightly within 24 h. Moreover, the study conclusion of the accuracy of different correction formulas only applies to the NCT used in our study. The selection of correction formulas in GAT and other NCT equipment should be based on further evidence and need additional explorations.

In summary, our study shows that the Shah correction formula of Pentacam is the most appropriate method for correcting postoperative IOP in patients undergoing SMILE surgery. In contrast, the Dresden, Orssengo-Pye, and Kohlhaas formulas are identified as the most suitable methods for obtaining reliable IOP values after FS-LASIK surgery. Our study may help guide the selection of appropriate IOP correction formulas after corneal refractive surgery.

## Supplementary Information

**Additional file 1.** 

## Data Availability

All data generated or analyzed during this study are included in this published article as the supplementary material.
